# Imaging of pelvic floor disorders involving the posterior compartment on dynamic MR defaecography

**DOI:** 10.4102/sajr.v28i1.2935

**Published:** 2024-10-11

**Authors:** Rajshree U. Dhadve, Karishma S. Krishnani, Tushar Kalekar, Eshan C. Durgi, Urvashi Agarwal, Suhas Madhu, Divyajat Kumar

**Affiliations:** 1 Department of Radiodiagnosis, Dr. D.Y. Patil Medical College, Hospital and Research Center, Dr D.Y. Patil Vidyapeeth, Pune, India

**Keywords:** pelvic floor dysfunction, posterior compartment disorders, anterior rectocoele, posterior rectocoele, rectal prolapse, enterocoele, MR defaecography

## Abstract

Disorders related to defaecation are widely common in the population and are often overlooked. Patients may present with a wide variety of symptoms such as constipation, faecal incontinence, painful defaecation, incomplete defaecation, hard stools, rectal bleeding, and mass per rectum. Complete clinical examination with radiological imaging, using dynamic MR Defaecography, can help in assessing, grading, and managing posterior compartment pelvic floor disorders and complex dysfunctions. The cases reveal a spectrum of pelvic floor disorders and complex dysfunctions including spastic pelvic floor syndrome, Grade 3 anorectal descent with rectocoele and cystocoele, tri-compartmental descent with obstructed defaecation syndrome, complete external rectal prolapse with Grade 3 abnormal anorectal descent and rectal intussusception.

**Contribution:**

This case series emphasises the importance of understanding the correlation of clinical and radiological imaging findings in posterior compartment pelvic floor dysfunctions through a series of cases presenting with clinical complaints related to defaecation.

## Introduction

Pelvic floor disorders represent a global health problem occurring in both genders. However, they are more common in women because of parity, menopause, age-related ligamentous laxity and complications related to childbirth. Literature has shown that pelvic floor disorders affect approximately 50% of women above 50 years of age.^1^

In the United States, the incidence rate is 1.5 to 1.8 per 1000 and is highest among women aged between 60 years and 69 years.^2^ In India, the incidence of genital organ prolapse is 5% to 8% in women who have delivered 1 or 2 children while it is 1.5% to 2% in nulliparous women.^3^

Approximately 20% of the women undergo surgery for pelvic floor disorders.^4^ The recurrence rate of pelvic organ prolapse after surgery is as high as 32%, likely because of failure to identify multi-compartment involvement before surgery. The reoperation rate is 11% to 20%.^5^

A wide range of clinical symptoms of the posterior compartment includes faecal incontinence, constipation, impaired evacuation (obstructed defaecation) and functional anal pain (levator ani syndrome).^6^ Concomitant symptoms associated with anterior and middle compartmental dysfunction, including vaginal or uterine prolapse, pain during sexual intercourse and urinary incontinence, may also be present. Multifactorial causes contribute to these disorders, involving anatomical and functional anomalies as well as gastrointestinal, psychological and chronic pain elements.

Dynamic MR defaecography is a multiphasic study with images obtained at rest, as well as during squeeze, strain and defaecation phases. The static images obtained at rest provide excellent soft tissue resolution, allowing better anatomical evaluation through its multiplanar capabilities. Dynamic phases provide a better functional evaluation of the pelvic floor. Dynamic MR defaecography can indicate additional findings that are not suspected from the history or clinical examination. It helps to accurately grade the disease, choose conservative versus surgical treatment and prevent failure rates after surgery.

Interpretation of posterior compartment disorders can sometimes be challenging because of the wide spectrum of abnormalities and complexity of the disorder, along with multi-compartmental involvement. However, comprehensive clinical and radiological evaluation helps to understand the pelvic floor anatomy and pathologies and also reduces the number of hospital visits. The imaging spectrum of some of the interesting posterior compartment abnormalities is documented in this case series.

## Ethical considerations

This article followed all ethical standards for research. The authors have followed the guidelines given by the institutional ethical committee for the publication of the case series. Written informed consent was obtained from the patients for publication along with the relevant images. Patients’ identities are not disclosed.

## Case Series

### Case 1

A male patient, aged 58 years, presented with a 1-year history of incomplete defaecation and constipation. He had undergone surgery for haemorrhoidectomy 10 years ago. During clinical examination, anal tone at rest, voluntary contraction (squeeze), and simulated contraction were assessed using digital rectal examination (DRE). Sphincter contraction was noticed during simulated daefecation. In addition, during examination of the puborectalis muscle while simulating defaecation, persistent puborectalis muscle contraction was observed, indicating a defaecation disorder. No abnormality was found on DRE at rest and MR defaecography was recommended.

**FIGURE 1:**
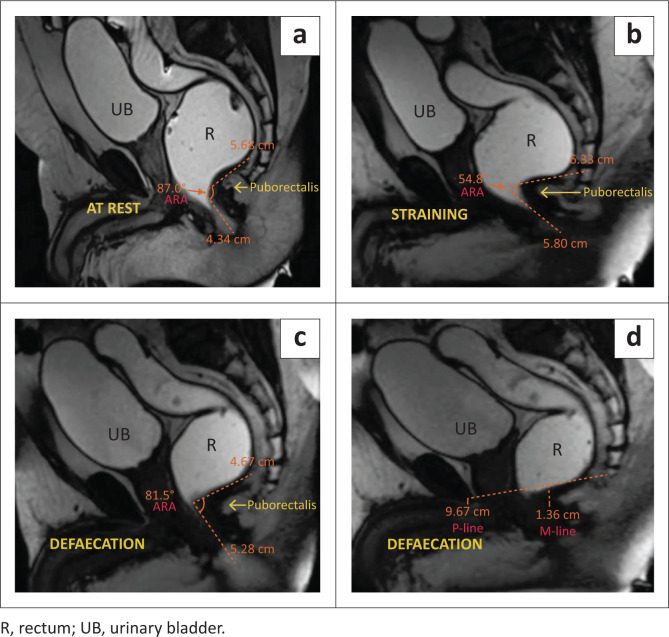
(a–d) MRI defaecogram: Spastic pelvic floor syndrome. (a) At rest shows the anorectal angle (ARA) at 87 degrees. (b) The straining phase shows a significant decrease in ARA to 54.8 degrees, with the puborectalis muscle indenting the anorectal junction. (c) Defaecation phase with an ARA of 81.5 degrees (less than that at rest), with persistent puborectalis indentation at the anorectal junction. (d) Defaecation phase showing Grade 1 anorectal descent.

On dynamic MR defaecography, the anorectal junction appeared to have migrated inferiorly by 1.6 cm, suggesting Grade 1 anorectal descent (Table 1). There was a reduction in the anorectal angle during the squeezing, straining, and defaecation phases. At rest, the anorectal angle was 87 degrees, significantly reducing to 54.8 degrees during straining and 81.5 degrees during defaecation. There was a lack of normal widening of the levator hiatus in the defaecation phase. The puborectalis sling was found to persistently indent the posterior anorectal junction in all phases (Figure 1), with incomplete evacuation of the rectally administered ultrasound jelly.

**TABLE 1:** Grading is performed by using the M-line and H-line for the measurement of pelvic floor relaxation. The H-line helps in identifying the hiatal widening and the M-line is used to measure the pelvic floor descent.

Grade	Severity	H-Line (cm)†	M-Line (cm)†
Grade 1	Normal	Less than 6	Less than 2
Grade 2	Mild	Between 6 and 8	Between 2 and 4
Grade 3	Moderate	Between 8 and 10	Between 4 and 6
Grade 4	Severe	More than 10	More than 6

*Source:* García del Salto L, De Miguel Criado J, Aguilera del Hoyo LF, et al. MR imaging–based assessment of the female pelvic foor. Radiographics. 2014;34(5):1417–1439. https://doi.org/10.1148/rg.345140137.

† , The H-line and M-line are measured on maximum strain in the mid-sagittal section.

Based on the clinical history and imaging findings of persistent indentation of the puborectalis muscle and failure of puborectalis muscle relaxation, a diagnosis of spastic pelvic floor syndrome was made.

### Case 2

A female patient, aged 67 years, presented with complaints of difficulty in defaecation, occasional faecal incontinence, and faecal soiling for the last 5 years to 6 years, with worsening symptoms in the past 2 years. Occasionally, manual digitation was required to initiate faecal evacuation. During the per-rectal examination, the patient was asked to perform the Valsalva manoeuvre, revealing anal descent, although the sphincter tone remained normal. A bulge was noted along the posterior vaginal wall during the examination.

**FIGURE 2:**
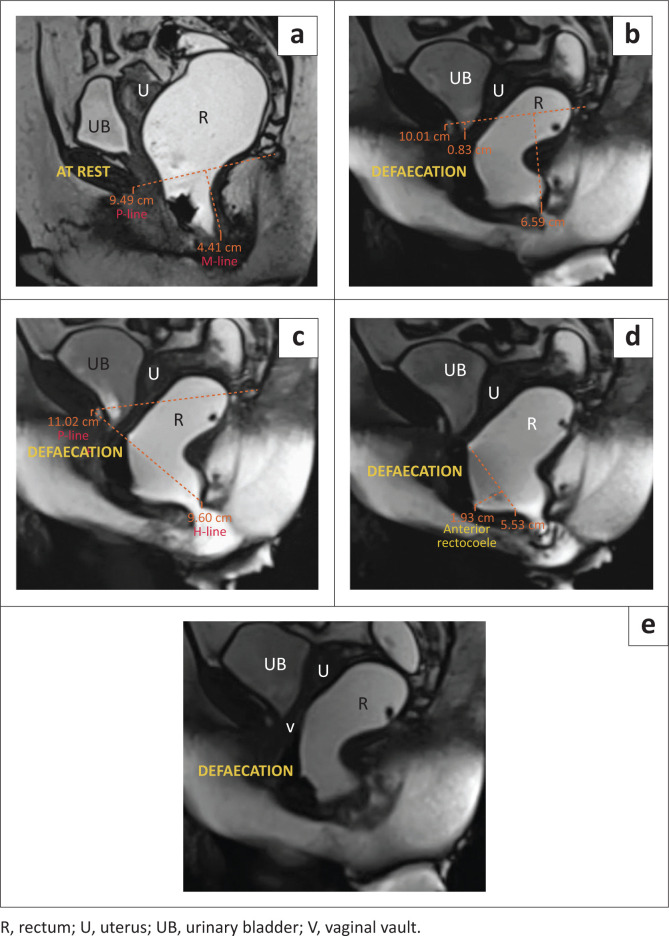
(a–e) MRI defaecogram: Grade 3 anorectal descent with anterior rectocoele. (a) At rest shows Grade 2 anorectal descent. (b) The defaecation phase shows Grade 3 anorectal descent with a small cystocoele. (c) During defaecation an increased H-line, representing levator hiatal widening is observed. (d) During defaecation a small anterior rectocoele is depicted. (e) The defaecation phase shows significant post-void residual intra-rectal jelly.

The MR defaecogram revealed abnormal anorectal descent, with the anorectal junction lying approximately 7.4 cm below the pubococcygeal line (PCL). An increased antero-posterior (AP) diameter of the levator hiatus was found, measuring 9.4 cm (H-line). A 3.1 cm bulge was found along the anterior rectal wall, indicating a medium-sized rectocoele. Significant post-void residue of rectal gel contrast, suggesting incomplete evacuation, was noticed at the end of the examination (Figure 2).

Based on the clinical and radiological findings, a diagnosis of Grade 3 abnormal anorectal descent (Table 2) with abnormal hiatal widening, a medium anterior rectocoele, a Grade 1 cystocoele (Table 2), and incomplete evacuation was made.

**TABLE 2:** Grading is performed by using the P-line as a reference for the measurement of pelvic organ prolapse.

Grade	Severity	The distance measured below the P-line (cm)
Grade 1	Mild	Between 1 and 3
Grade 2	Moderate	Between 3 and 6
Grade 3	Severe	More than 6

*Source:* García del Salto L, De Miguel Criado J, Aguilera del Hoyo LF, et al. MR imaging–based assessment of the female pelvic foor. Radiographics. 2014;34(5):1417–1439. https://doi.org/10.1148/rg.345140137.

P-line, pubococcygeal line.

### Case 3

A female patient, aged 40 years, presented with complaints of hard stools and chronic constipation for 1 year. She reported a history of mass per rectum during defaecation, which reduced spontaneously. There was no history of rectal bleeding. In addition, she complained of occasional itching around the perianal region.

A bimanual rectovaginal examination revealed a bulge along the posterior vaginal wall, which increased in size during straining. Rectal examination indicated rectal descent, with skin tags observed at the 12 o’clock and 6 o’clock positions. Abdominal and pelvic ultrasound results were normal.

**FIGURE 3:**
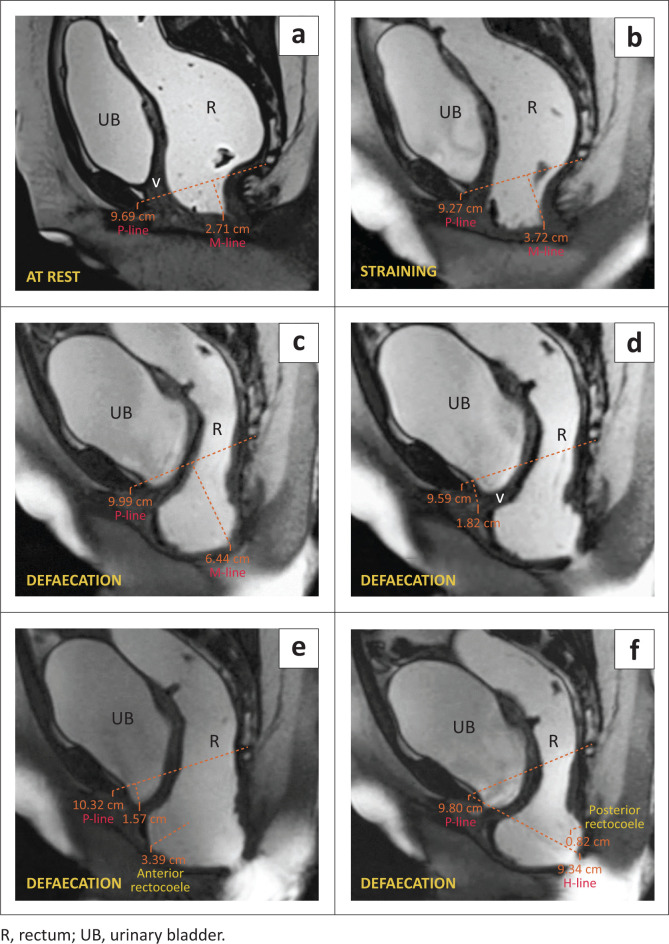
(a–f) MRI defaecogram: Obstructed defaecation with Grade 3 anorectal descent, anterior and posterior rectocoele. (a) At rest, (b) on straining, (c) during defaecation, shows progressive Grade 1, 2, and 3 anorectal descent. (d) During defaecation, mild vaginal vault prolapse was seen. (e) Defaecation phase demonstrates a mild cystocoele and medium anterior rectocoele. (f) The defaecating sequence shows a small posterior rectocoele with an increased H-line representing hiatal widening.

Further evaluation with an MR defaecogram was conducted. At rest, the anorectal junction was 2.1 cm below the PCL, indicating Grade 1 anorectal descent (Table 2). Progressive increases in descent were observed during straining (approximately 3.72 cm, Grade 2) and defaecation (6.4 cm, Grade 3). During defaecation, an abnormal bulge along the anterior rectal wall measuring 3.4 cm in AP diameter, suggested a medium-sized anterior rectocoele. In the late defaecation phase, a small bulge was observed along the posterior rectal wall suggestive of a small posterior rectocoele. An abnormal increase in the H-line (9.34 cm) during the defaecation phase suggested levator hiatus widening. Significant post-void residual jelly was found at the end of defaecation. In addition, the urinary bladder was observed approximately 1.5 cm below the PCL, and the vaginal vault was seen 1.8 cm below the line (Figure 3).

The final diagnosis was Grade 3 anorectal descent (Table 2) with moderate hiatal widening (Table 1), obstructed defaecation with a medium anterior rectocoele, small posterior rectocoele, Grade 1 cystocoele, and vaginal vault prolapse (Table 2). The posterior rectocoele, cystocoele, and vaginal vault prolapse, which were not identified during clinical examination, were detected during MRI defaecography. This discovery will significantly impact surgical management.

### Case 4

A female patient, aged 34 years, presented with complaints of a mass per rectum, persisting for 2 years. She had a history of two full-term normal vaginal deliveries.

Clinical examination revealed complete rectal prolapse. Vaginal examination showed a mild bulge along the posterior vaginal wall. Surgical correction was advised and a preoperative MR defaecogram was performed.

**FIGURE 4:**
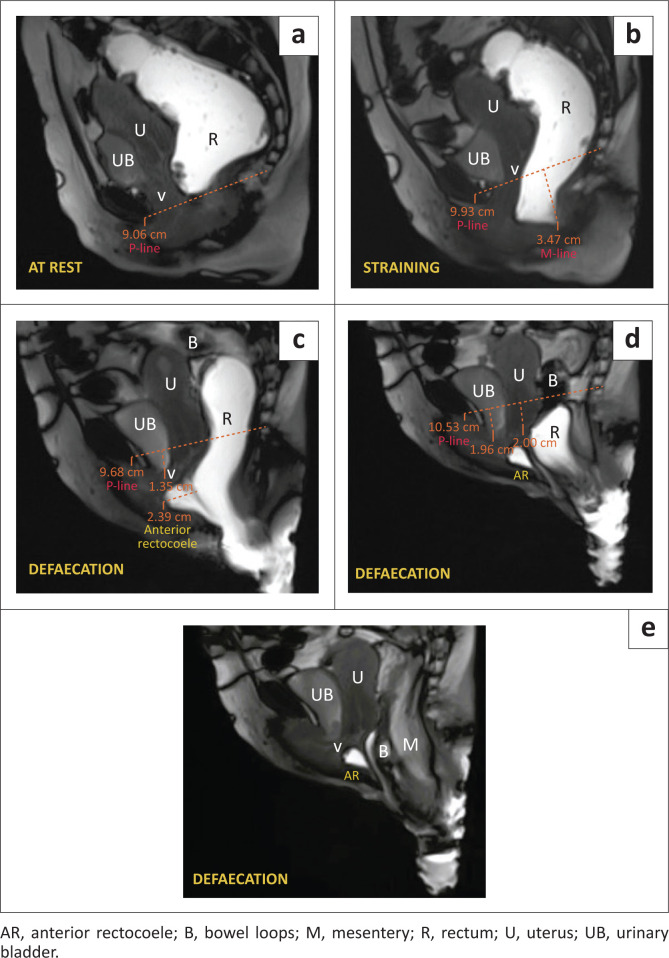
(a–e) MRI defaecogram: Rectal prolapse with enterocoele and anterior rectocoele (a) at rest. (b) Straining phase showing Grade 2 anorectal descent. (c) Defaecation phase depicting medium anterior rectocoele and small cystocoele. (d) Defaecation phase with tricompartment involvement, showing small cystocoele, Grade 1 uterine prolapse, rectum lying below the pubococcygeal line (P-line), bowel loops descending below the P-line and anterior rectocoele with retained jelly. (e) Defaecation phase showing complete rectal prolapse and further descent of the bowel loops (B) and mesentery (M) outside the anal verge.

As demonstrated in Figure 4, MRI findings revealed an increased AP diameter of the hiatus (H-line: 14 cm) at rest, suggesting severe levator hiatal widening. During evacuation sequences, global pelvic floor descent was observed. The anorectal junction was positioned approximately 6.4 cm below the PCL at rest, migrating inferiorly by 11 cm during the defaecation sequences. A complete external prolapse of the rectum was evident. The anterior rectal wall exhibited abnormal bowing measuring 2.7 cm on straining and defaecation images. Bowel loops, along with the mesentery, descended into the cul-de-sac during the defaecating sequences, extending beyond the anal verge, suggestive of enterocoele. The urinary bladder neck was situated 2.8 cm below the PCL, and the vaginal vault was positioned 2.6 cm below the PCL.

A diagnosis of complete external rectal prolapse with Grade 3 anorectal descent, along with mild anterior rectocoele (AR), Grade 1 cystocoele, Grade 1 uterine prolapse, and severe enterocoele, was established (Table 2).

### Case 5

A male patient, aged 44 years, presented with complaints of chronic constipation and difficulty in defaecation, requiring manual evacuation for the past 5 years, along with a history of haemorrhoids for 9 years.

On per-rectal examination, anorectal descent was observed, with a bulge along the anterior rectal wall, indicating AR. Haemorrhoids were found at the 12 o’clock position. The patient underwent an ultrasound of the abdomen and pelvis and colonoscopy revealed no obvious abnormalities.

**FIGURE 5:**
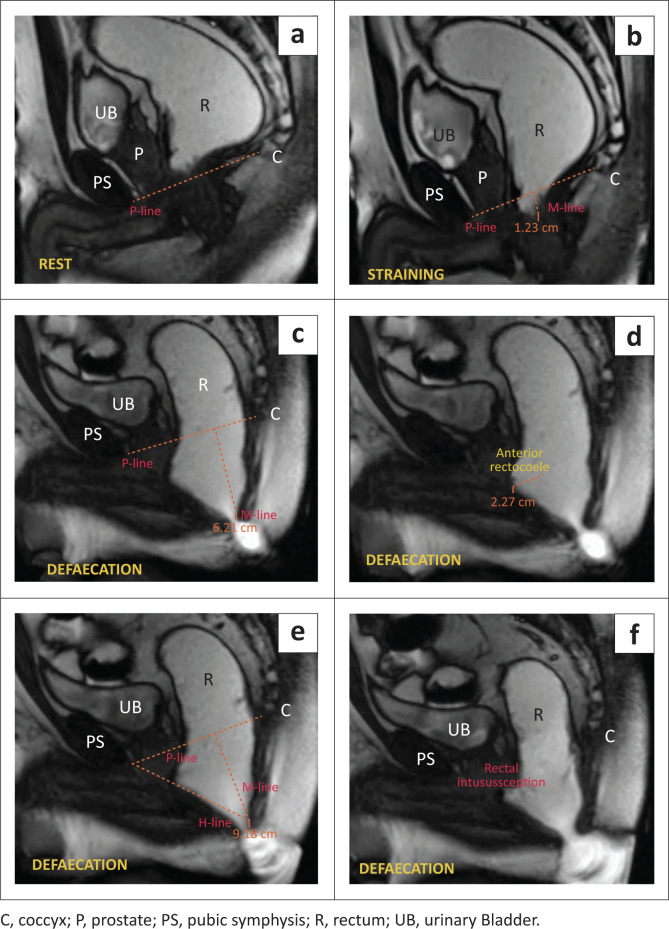
(a–e) MRI defaecogram: Rectal intussusception. (a) At rest showed no abnormality. (b) On straining showed anorectal descent with the anorectal junction 1.2 cm below the P-line. (c) The defaecation phase revealed the anorectal junction 6.2 cm below the P-line, representing anorectal descent. (d) Defaecation phase reveals a moderate anterior rectocoele (2.27 cm). (e) The defaecation phase shows an increased H-line (9.18 cm), depicting levator hiatal widening. (f) The defaecating sequence also shows recto-rectal intussusception.

The MRI defaecography (Figure 5a–g) showed no abnormalities at rest. During the straining phase, mild anorectal descent was found, with the anorectal junction positioned 1.6 cm below the P-line. In the defaecation sequence, the anorectal junction was 6.21 cm below the P-line, indicating anorectal descent. There was abnormal widening of the levator hiatus (H-line: 9.18 cm) along with an AR measuring 2.27 cm. Towards the end of the defaecation phase, telescoping of the rectal walls into the rectum was observed, denoting rectal intussusception. Significant post-void residual jelly was present at the end of defaecation.

A diagnosis of Grade 3 anorectal descent (Table 2), with abnormal moderate levator hiatal widening (Table 1), moderate AR, and recto-rectal intussusception was made based on the clinical and radiological findings. These findings significantly impacted the management of the patient.

## Discussion

Dynamic MR defaecography is a novel, non-invasive technique for the evaluation of pelvic floor abnormalities. The technique provides both anatomical and functional information of the pelvic floor structures, including the urinary bladder, uterus, vagina, rectum and anal canal. In addition, it allows for the study of the puborectalis and levator muscles, as well as analysis of the anorectal angle (ARA) and the opening of the anal canal. Excellent soft tissue resolution provides important additional anatomical details, which are crucial, especially during surgery, for example, thinning of the puborectalis muscles.^7^

Up to 17% of the global population worldwide is suffering from chronic constipation and 50% are diagnosed with outlet obstruction constipation.^8^ Therefore, approximately half of the patients with pelvic floor abnormality involving the posterior compartment have functional disease, which can be diagnosed by MR defaecography. Common causes of outlet obstruction constipation are rectocoele, dyssynergic defaecation, rectoanal intussusception and enterocoele.^8^

Urinary, genital, and anorectal organs are intricately related to each other in the functional and structural support as they traverse the puborectalis fascia. Therefore, although patients may present with only posterior compartment symptoms, 95% pelvic floor dysfunctions involve abnormalities in all three compartments.^8^

This case series aims to showcase basic posterior compartment abnormalities in patients who only had complaints related to this compartment. Although patients did not exhibit symptoms related to other compartments, it is very common to observe involvement of other compartments simultaneously.^9^ This is where the role of MRI defaecography proves extremely beneficial and is increasingly utilised.

**FIGURE 6:**
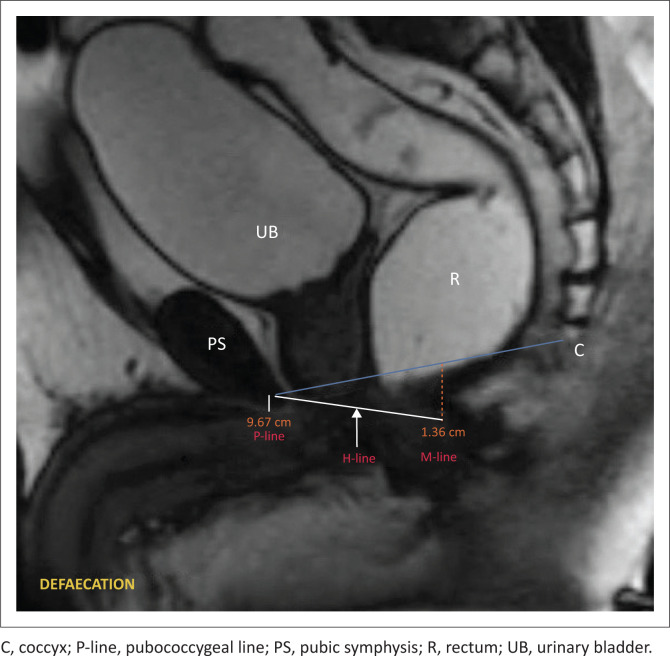
MR defaecogram during the defaecating phase, illustrating reference lines. P-line (blue line), M-line (red line), and H-line (white line).

Interpretation of MR defaecography can be conducted using reference lines, namely the PCL (P-line), M-line, and H-line (Figure 6).

On dynamic MRI, the PCL (P-line) is delineated on the midsagittal plane to radiologically define the pelvic floor level.^10^ This line is constructed by connecting the inferior rim of the pubic symphysis to the most inferior sacrococcygeal joint. In healthy individuals, the levator plate aligns parallel to the PCL. The H-line and M-line are additional reference lines employed to identify pelvic floor prolapse and relaxation.^10^ On a midsagittal image, an H-line can be drawn from the lowest margin of the pubic symphysis to the anorectal junction, representing the anteroposterior diameter of the levator hiatus. A line drawn perpendicularly from the PCL to the furthest distal point of the H-line is termed the M-line, indicating the descent of the levator hiatus from the PCL.

### Spastic perineum syndrome

Normally during defaecation, the puborectalis muscle relaxes, causing widening of the anorectal angle (ARA). Spastic perineum syndrome is a functional pelvic floor disorder, also known as paradoxical contraction of the puborectalis muscle, anismus, or pelvic floor dyssynergia.^11^ It is characterised by involuntary and paradoxical contraction of the puborectalis muscle, leading to a lack of normal widening of the ARA during the evacuation phase.^9,12^ The puborectalis muscle may appear hypertrophied. Sometimes, it may or may not be associated with pelvic floor descent. Anorectal manometry typically reveals elevated pressures in this condition.

### Rectocoele

A rectocoele, characterised by the protrusion of the rectal wall beyond its usual morphology, primarily arises from diminished muscle tone in the pelvic floor muscles and the relaxation of the rectovaginal fascia. Factors contributing to the development of a rectocoele encompass events such as vaginal delivery, the application of forceps during childbirth, persistent straining during defaecation, surgical hysterectomy, the natural process of ageing, and weakened pelvic musculature.^7^ Clinical manifestations typically entail vaginal bulging, dyspareunia, and the perception of a vaginal mass. The manifestation of symptoms associated with a rectocoele significantly impacts individuals, often resulting in constipation, incomplete bowel movements, and obstructive complications. Defaecography can help diagnose rectocoeles by visualising the rectum during straining or defaecation. Defaecography can also evaluate the emptying dynamics of a rectocoele: Retention of the contrast material seen at the end of defaecation is suggestive of incomplete evacuation.

The protrusion of the rectal wall beyond the normal margin is measured to determine the extent of the rectocoele. Rectocoeles are classified as small (< 2 cm), medium (2 cm – 4 cm), and moderate (> 4 cm).^13^ Rectocoeles that are larger than 2 cm, do not empty during defaecation, or cause symptoms, are considered abnormal. Up to 80% of patients with small rectocoeles are asymptomatic. Rectocoeles can be anterior or posterior, depending on the contour. Anterior rectocoeles are more common.^6,9^

### Rectal descent

Rectal descent refers to the downward displacement of the anorectal junction below the PCL. Frequently, an anomalous descent of the pelvic floor towards the rear is linked with the engagement of both the anterior and middle compartments, indicating a generally prevalent pelvic floor weakness. The condition known as descending perineal syndrome manifests as broad pelvic floor weakness. Initially, manifestations of this syndrome predominantly encompass constipation and perineal discomfort; however, with time, the condition progresses to exacerbate faecal and urinary incontinence.^7^ Aetiological factors associated with the syndrome encompass pudendal nerve impairments following childbirth trauma or neuropathy, as well as prolonged straining.^14^ In certain cases, asymptomatic individuals may present with a minor descent of less than 3 cm. While abnormal rectal descent may manifest at rest, it typically occurs during defaecation or straining.

### Rectal intussusception

Rectal intussusception results in the folding and telescoping of the rectal walls into the rectum or anal canal while defaecating.^15,16^ This causes mechanical obstruction leading to incomplete evacuation. Patients usually present with non-specific symptoms related to defaecation. During MR defaecography, rectal intussusception is typically seen towards the end of evacuation. The location of rectal intussusception can be categorised into three groups: intra-rectal, intra-anal and extra-anal.

It is crucial to distinguish between full rectal wall intussusception and mucosal invagination alone because the surgical approach varies significantly. At this juncture, MR defeacography stands out as the sole imaging examination capable of precisely discerning the two conditions.^11^

### Rectal prolapse

Rectal prolapse occurs when the rectal wall extends through the anal orifice. Initially, intra-rectal intussusception is observed, which later progresses to complete prolapse.^8^ Symptoms include constipation, incomplete bowel evacuation, accidental stool leakage, and bleeding from ulcers in the rectum. Rarely, untreated cases of rectal prolapse can lead to strangulation.

### Levator hiatal widening

The levator hiatus is a large potential opening in the human body between the pubic bone and the levator ani muscle. During straining or defaecation, the hiatus relaxes and widens both radially and downwards. The degree of radial relaxation is measured by looking at the length of the H-line and the degree of downwards relaxation is measured by evaluating the M-line.^7^

### Enterocoele

An enterocoele is a condition where the peritoneum, which has mesenteric fat, small intestine, or parts of the sigmoid colon, protrudes and pushes into the back wall of the vagina. It usually occurs in women with a history of hysterectomy, leading to separation of the fascia between the anterior and posterior vagina. Symptoms vary depending on the extent and size of the enterocoele but may include a feeling of heaviness in the vagina, chronic constipation, or incomplete defaecation. Magnetic resonance defaecography can be used to visualise the condition, showing an enlargement of the recto-genital fossa and abnormal descent of fat, small bowel, or sigmoid colon. This can be more noticeable when the patient strains or performs a manoeuvre called Valsalva. However, traditional defaecography may not always detect an enterocoele if the small bowel, sigmoid colon, or peritoneal cavity is not adequately visualised.^7^

As we can infer from the above-mentioned cases, anorectal descent was noticed in all patients and AR was seen in three patients. These are among the most commonly observed pelvic floor pathologies.^8,17^ Associated levator hiatal widening, which is measured by the H-line, was seen in three of the patients. The posterior rectocoele is seen as a bulge in the posterior wall of the rectum and is less common than the AR. Of the four cases presented, only one patient presented with a posterior rectocoele. Enterocoele is another finding that is not commonly encountered,^8^ but it is an easily overlooked pathology. Therefore, it is important to examine the descent of the bowel loops in the recto-genital fossa or beyond it, as demonstrated in Case 4. Case 1 presented a classic example of spastic perineum syndrome, characterised by a reduction of the ARA in the straining and defaecation phases, which points to the inability of the pelvic floor to relax during the straining and defaecation phases. It can be due to multiple causes, one of which, as demonstrated in Case 1, was persistent indentation of the puborectalis muscle. While major findings were diagnosed clinically, associated pathologies and mild abnormalities were not detected during clinical examination, however, were successfully diagnosed through dynamic MRI evaluation.

## Conclusion

Pelvic floor dysfunction is a major underestimated public health issue around the world, especially in women. Most often, surgical treatment is the only option. Static and dynamic magnetic resonance defaecography serves as a comprehensive tool for evaluating the posterior compartment, with the added advantage of assessing all three compartments in one sitting. For conditions affecting multiple pelvic compartments, MRI is the imaging method of choice. It can guide the surgical procedure, enhancing postoperative outcomes and reducing recurrence rates because of overlooked pathologies. It is a non-invasive assessment, which can be utilised to diagnose pelvic floor problems without the risk of exposure to ionising radiation. Often, patients presenting with symptoms involving the posterior compartment have associated multi-compartment involvement.

## References

[1] Nijland DM, Van Genugten LT, Dekker KS, et al. The added value of conventional defecography and MRI defecography in clinical decision making on treatment for posterior compartment prolapse. Int Urogynecol J. 2023;34(2):507–515. 10.1007/s00192-022-05181-x 35403883 PMC9870817

[2] Wang B, Chen Y, Zhu X, et al. Global burden and trends of pelvic organ prolapse associated with aging women: An observational trend study from 1990 to 2019. Front Public Health. 2022;10:975829. 10.3389/fpubh.2022.97582 36187690 PMC9521163

[3] Ryan GA, Purandare NC, Ganeriwal SA, et al. Conservative management of pelvic organ prolapse: Indian contribution. J Obstet Gynecol India. 2021;71:3–10. 10.1007/s13224-020-01406-5 33814793 PMC7960828

[4] Jha P, Sarawagi R, Malik R, et al. Static and dynamic magnetic resonance imaging in female pelvic floor dysfunction: Correlation with pelvic organ prolapse quantification. Cureus. 2023;15(9):e44915. 10.7759/cureus.44915 37814774 PMC10560544

[5] Lallemant M, Clermont-Hama Y, Giraudet G, et al. Long-term outcomes after pelvic organ prolapse repair in young women. J Clin Med. 2022;11:6112. 10.3390/jcm11206112 36294437 PMC9605202

[6] Pfeifer J, Oliveira L, Park UC, Gonzalez A, Agachan F, Wexner SD. Are interpretations of video defecographies reliable and reproducible? Int J Colorectal Dis. 1997;12:67–72. 10.1007/s003840050083 9189773

[7] García del Salto L, De Miguel Criado J, Aguilera del Hoyo LF, et al. MR imaging–based assessment of the female pelvic foor. Radiographics. 2014;34(5):1417–1439. 10.1148/rg.345140137 25208288

[8] Li M, Jiang T, Yang X, et al. Association of compartment defects in anorectal and pelvic floor dysfunction with female outlet obstruction constipation by dynamic MR defecography. Eur Rev Med Pharmacol. 2015;19:1407–1415. 25967716

[9] Darwish HS, Zaytoun HA, Kamel HA, et al. Assessment of pelvic floor dysfunctions using dynamic magnetic resonance imaging. Egypt J Radiol Nucl Med. 2014;45(1):225–229. 10.1016/j.ejrnm.2013.12.006

[10] Singh K, Jakab M, Reid WM, Berger LA, Hoyte L. Three dimensional magnetic resonance imaging assessment of levator ani morphologic features in different grades of prolapse. Am J Obstet Gynecol. 2003;188(4):910–915. 10.1067/mob.2003.254 12712085

[11] Salvador JC, Coutinho MP, Venâncio JM, Viamonte B. Dynamic magnetic resonance imaging of the female pelvic floor – A pictorial review. Insights Imaging. 2019;10:1–6. 10.1186/s13244-019-0687-9 30689115 PMC6352388

[12] Roos JE, Weishaupt D, Wildermuth S, Willmann JK, Marincek B, Hilfiker PR. Experience of 4 years with open MR defecography: Pictorial review of anorectal anatomy and disease. Radiographics. 2002;22(4):817–832. 10.1148/radiographics.22.4.g02jl02817 12110712

[13] Thapar RB, Patankar RV, Kamat RD, Thapar RR, Chemburkar V. MR defecography for obstructed defecation syndrome. Indian J Radiol Imaging 2015;25(01):25–30. 10.4103/0971-3026.150134 25709162 PMC4329684

[14] Dimitriou N, Shah V, Stark D, Mathew R, Miller AS, Yeung JM. Defecating disorders: A common cause of constipation in women. Women’s Health. 2015;11(4):485–500. 10.2217/WHE.15.25 26238417

[15] Felt-Bersma RJ, Cuesta MA. Rectal prolapse, rectal intussusception, rectocele, and solitary rectal ulcer syndrome. Gastroenterol Clin North Am. 2001;30(1):199–222. 10.1016/S0889-8553(05)70174-6 11394031

[16] Kenton K, Shott S, Brubaker L. The anatomic and functional variability of rectoceles in women. Int Urogynecol J Pelvic Floor Dysfunct. 1999;10(2):96–99. 10.1007/PL00004019 10384970

[17] Takahashi T, Yamana T, Sahara R, et al. Enterocele: What is the clinical implication? Dis Colon Rectum. 2006;49(1):S75–S81. 10.1007/s10350-006-0683-2 17106819

